# Skeletal Muscle Satellite Cells, Mitochondria, and MicroRNAs: Their Involvement in the Pathogenesis of ALS

**DOI:** 10.3389/fphys.2016.00403

**Published:** 2016-09-13

**Authors:** Stavroula Tsitkanou, Paul A. Della Gatta, Aaron P. Russell

**Affiliations:** ^1^Athletics Laboratory, School of Physical Education and Sport Science, University of AthensAthens, Greece; ^2^School of Exercise and Nutrition Sciences, Institute for Physical Activity and Nutrition (IPAN), Deakin UniversityGeelong, VIC, Australia

**Keywords:** skeletal muscle, amyotrophic lateral sclerosis, satellite cell, mitochondria, miRNA, neuromuscular junction

## Abstract

Amyotrophic lateral sclerosis (ALS), also known as motor neuron disease (MND), is a fatal motor neuron disorder. It results in progressive degeneration and death of upper and lower motor neurons, protein aggregation, severe muscle atrophy and respiratory insufficiency. Median survival with ALS is between 2 and 5 years from the onset of symptoms. ALS manifests as either familial ALS (FALS) (~10% of cases) or sporadic ALS (SALS), (~90% of cases). Mutations in the copper/zinc (CuZn) superoxide dismutase (SOD1) gene account for ~20% of FALS cases and the mutant SOD1 mouse model has been used extensively to help understand the ALS pathology. As the precise mechanisms causing ALS are not well understood there is presently no cure. Recent evidence suggests that motor neuron degradation may involve a cell non-autonomous phenomenon involving numerous cell types within various tissues. Skeletal muscle is now considered as an important tissue involved in the pathogenesis of ALS by activating a retrograde signaling cascade that degrades motor neurons. Skeletal muscle heath and function are regulated by numerous factors including satellite cells, mitochondria and microRNAs. Studies demonstrate that in ALS these factors show various levels of dysregulation within the skeletal muscle. This review provides an overview of their dysregulation in various ALS models as well as how they may contribute individually and/or synergistically to the ALS pathogenesis.

## Pathophysiology of ALS

**Figure 1:**
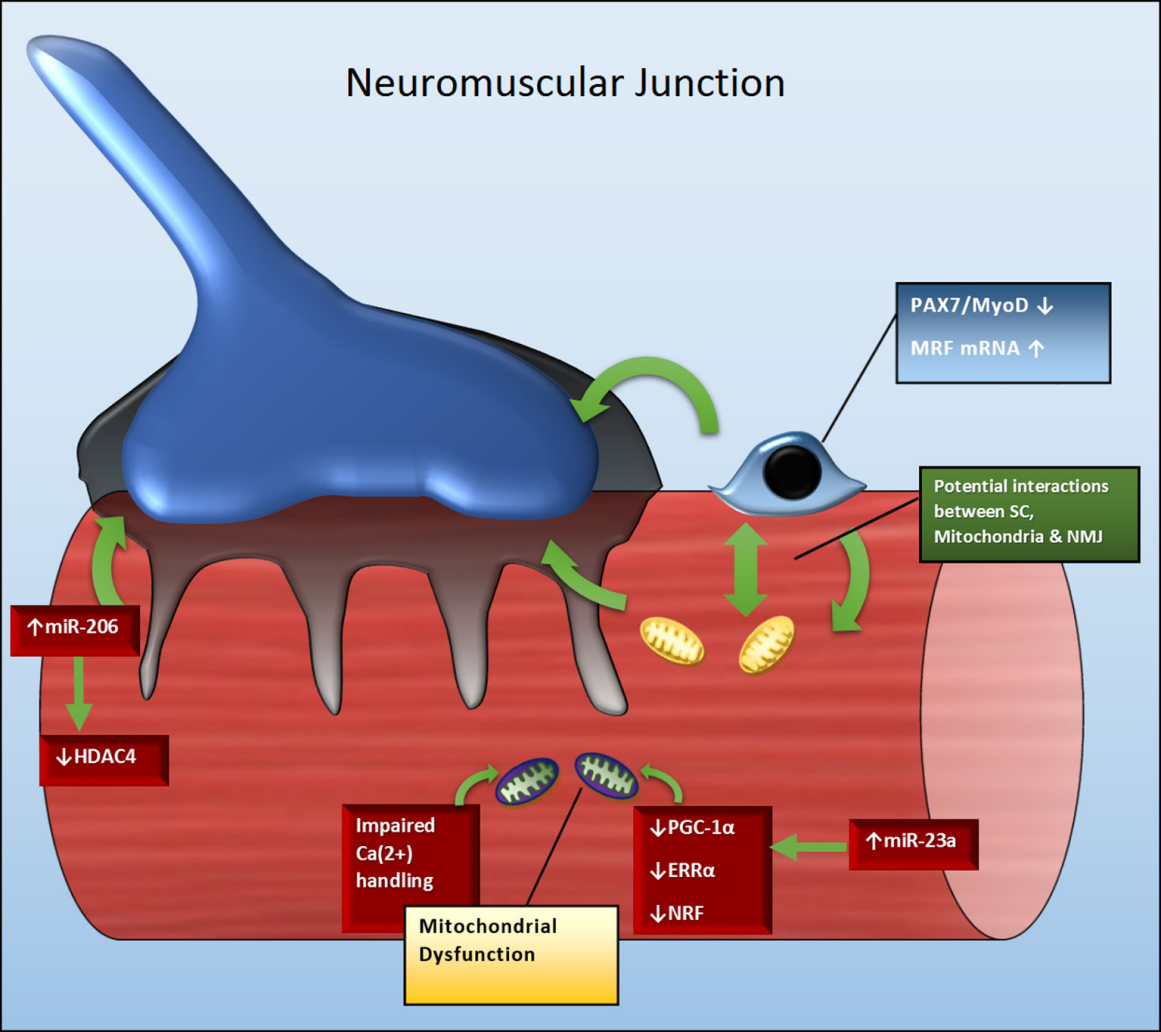
**Potential crosstalk between SC, mitochondria and miRNAs to influence NMJ and skeletal muscle health in ALS**. Green arrows indicate regulatory relationships between molecules, as well as between molecules and structures. A reduction in Pax7 and MyoD may contribute to the attenuation in SC activation and number. SCs not only maintain skeletal muscle fiber size and function, but those located near the NMJ also play role in maintaining NMJ stability. Similarly, downregulation of molecular targets including PGC-1α, ERRα, and NRF may impair mitochondrial biogenesis and function. As mitochondria play role in maintaining calcium homeostasis and muscle repair, their dysregulation may also contribute to skeletal muscle and NMJ deterioration. Perturbations in miRNAs, such as miR-206, may negatively impact regenerative capacity and NMJ stability via their control over MRFs (myogenic regulatory factors) and HDAC4 transcription. Additionally, the upregulation of miRNA-23a may negatively impact the PGC-1α signaling cascade and mitochondrial function; as consequence indirectly contributing to impaired skeletal muscle repair and NMJ stability.

In ALS the cause of motor neuron degeneration remains equivocal. However, motor neuron death is a leading candidate (Kennel et al., 1996). Numerous mechanisms are suggested to be involved in ALS. Therefore, it is considered a “multisystemic” disease in which changes in structural, physiological, and metabolic parameters in different cell types, may act mutually and synergistically, to contribute to disease on set and severity (Cozzolino et al., 2008; Musaro, 2013). Several factors have been investigated as possible contributors to motor neuron death, including excitotoxicity, oxidative stress, deficits in axonal transport, neurofilament aggregation, protein aggregation, involvement of non-neuronal cells, mitochondrial dysfunction and the dysregulation in microRNA processing and expression. The motor neuron degeneration observed in ALS is a cell non-autonomous phenomenon involving numerous cell types. Evidence that ALS pathology starts at the neuromuscular junction (NMJ) rather than the motor neuron (Fischer et al., 2004; Jokic et al., 2006; Dupuis et al., 2009) has led to ALS being recognized and reviewed as a distal axonopathy, whereby skeletal muscle contributes to a retrograde signaling cascade that degrades motor neurons (Dupuis et al., 2009; Dadon-Nachum et al., 2011; Boyer et al., 2013; Moloney et al., 2014). Disturbances in several mechanisms important for maintaining healthy skeletal muscle mass and function, such as satellite cell activity, mitochondrial biogenesis and miRNA regulation, are observed in ALS. Interestingly, these factors may act synergistically to maintain healthy skeletal muscle and may influence NMJ number and activity. This review highlights our understanding of how satellite cell activation, mitochondrial function and miRNAs may impact ALS pathogenies with a focus on skeletal muscle and NMJ degeneration (Figure 1 and Table 1).

**Table 1:** **Summary of changes in skeletal muscle satellite cells, MRFs, mitochondria and microRNAs**.

	**Disease progression in SOD^G93A^ mice**				**ALS patients**
	**P40**	**P60**	**P90**	**P120**	
Satellite cells	↓ In satellite cell number both in slow and fast fibers (Manzano et al., 2013)	↑ In satellite cell number in slow fibers and ND in satellite cell number in fast fibers (Manzano et al., 2013)	↓ In satellite cell number in slow fibers and ↑ in satellite cell number in fast fibers (Manzano et al., 2013)	↑ In satellite cell number in slow fibers and ND in satellite cell number in fast fibers (Manzano et al., 2013)	Muscle tissue: ND in satellite cell number ↑ Co staining of MyoG/Pax7 ↓ Co-staining of MyoD/Pax7, Myf5. (Pradat et al., 2011)
MRFs	ND in Pax7, Myf5, Myod1 and Myog mRNA. ND in Pax7, MYF5, MYOD1, MYOG protein (Manzano et al., 2013)	↑ Pax7 and Myog mRNA. ↑ MYF5 protein (Manzano et al., 2013)	↑ Myf5, Myod1 and Myog mRNA. ↑ Pax7, MYF5, MYOD1 protein (Manzano et al., 2013)	↑ Pax7, Myf5, Myod1 and Myog mRNA. ND in Pax7, MYF5, MYOD1 and MYOG protein (Manzano et al., 2013)	Myoblast cultures: ↑ MyoD mRNA, ND Pax7 mRNA, (Scaramozza et al., 2014). Myotube cultures: ↓ MyoG protein (Scaramozza et al., 2014)
Mitochondria	↓ PGC-1a, NRF-1, Tfam, mnSOD mRNA. ND in PGC-1a, NRF-1, Tfam, mnSOD, AChRε protein (Thau et al., 2012)	↓ migration rate of mt-PAGFP (↓ mt-fusion and fission). ND in mt-morphology. Mt-membrane depolarization. Fragmentation of the mt-network (Dupuis et al., 2004; Faes and Callewaert, 2011; Bozzo et al., 2016)	↓ PGC-1a, PGC-1β, ERRa, NRF-1, Mfn1, Mfn2 and COXIV mRNA (Russell et al., 2012) ↓ PGC-1a and Utrn mRNA. ND in PGC-1a, NRF-1, Tfam, mnSOD, AChRε protein (Thau et al., 2012)	↓ PGC-1a, PGC-1β, ERRa, NRF-1, Mfn1, Mfn2 and COXIV mRNA (Russell et al., 2012) ↓ PGC-1a, NRF-1, Tfam, mnSOD mRNA. ↓ NRF-1 protein (Thau et al., 2012)	↓ NADH:CoQ oxidoreductase, cytochrome c oxidase (COX), mitochondrial DNA and mitochondria Mn-SOD (Wiedemann et al., 1998; Vielhaber et al., 2000) ↓ PGC-1a, PGC-1β, ERRa, NRF-1, Mfn1, Mfn2 and COXIV mRNA. ↓ PGC-1a, Mfn1 and COXIV protein. ND in Mfn2 and NRF-1. ↓ CS and COX activity (Russell et al., 2012) ↓ PGC-1a, NRF-1, NRF-2, Utrn mRNA. ND in PGC-1a, NRF-1, Tfam, mnSOD protein (Thau et al., 2012)
miRNAs	ND in miR-206 (at post-natal age of 1 month) (Williams et al., 2009)			↑ miR-206 (at post-natal age of 8 months). ND in miR-206 (at post-natal of 5 months) (Williams et al., 2009)	↑ miR-23a,-29b,-31,-206,-455. (Russell et al., 2012)

*P40, post-natal age of 40 days; MRF, myogenic regulatory factor; ND, no significant difference compared to control group; mt, mitochondrial*.

## The role of satellite cells in ALS pathology

Overexpressing the mutant SOD1 protein specifically in healthy skeletal muscle induces an ALS phenotype and a degradation of motor neurons (Dobrowolny et al., 2008; Wong and Martin, 2010), supporting a direct role of skeletal muscle in disease progression. However, the mechanisms within the muscle that contribute to the initial loss of muscle mass and eventual degeneration of the NMJ and motor neurons are unknown. Skeletal muscle repairs itself via the well-coordinated process of myogenesis that relies on efficient satellite cell (SC) activation, proliferation, fusion, and differentiation (Mauro, 1961). Under basal conditions SCs are quiescent, becoming activated in response to acute injury, muscle denervation or exercise stimuli. Once activated, SC's proliferate, differentiate into myoblasts and fuse with existing myofibres resulting in muscle fiber repair and/or growth. When SCs are mitotically quiescent, they express the myogenic regulatory factor (MRF) family member, paired box 7 transcription factor, Pax7 (Seale et al., 2000) as well the adhesion molecule M-cadherin (Irintchev et al., 1994), the hematopoietic progenitor cell antigen, CD34 (Beauchamp et al., 2000) and another MRF, myogenic transcription factor-5, Myf5 (Tajbakhsh et al., 1997). Upon activation, and when the cells are no longer in the quiescent state, SCs also express the MRF, myogenic differentiation 1 (MyoD) (Fuchtbauer and Westphal, 1992), however CD34 is not expressed (Zammit et al., 2006). Therefore, co-expression of both MRF family members, Pax7 and MyoD, is an indication that the cells have moved from the quiescent to the active state. As the activated cells begin to proliferate, Pax7 is downregulated, while MyoD and/or Myf5 expression remains. During differentiation, another MRF, myogenin (MyoG; Myf4) is expressed and is essential for SC differentiation into multinucleated myotubes. As such, myogenin is used as a marker of the onset of myogenic differentiation (Fuchtbauer and Westphal, 1992). The myogenic capacity of skeletal muscle from either patients with ALS or rodent models of human ALS has not been extensively studied so our understanding of how myogenic perturbations contributes to disease on-set and progression is limited.

Satellite cell cultures obtained from ALS patient biopsies have been observed to proliferate similarly to cultures obtained from healthy muscle, however, with a morphology that resembles senescent cells (Pradat et al., 2011). In contrast, another study has observed that ALS patient-derived myoblast cultures proliferate faster than control cultures (Scaramozza et al., 2014). Compared with control cultures, the ALS patient-derived myoblast cultures had significantly more MyoD mRNA, but similar Pax7 mRNA, levels suggesting that the ALS patient-derived myoblast cultures were in a more committed state (Scaramozza et al., 2014). These myogenic markers were not measured by Pradat et al. (2011). The different observations made by Pradat et al. (2011) and Scaramozza et al. (2014), when comparing ALS-derived myoblast proliferation with control myoblast proliferation, may be attributed to different muscle samples biopsied, disease stage when the biopsies were taken as well as patient age. However, both of these studies observed that ALS-derived myoblasts are unable to fully differentiate into myotubes (Pradat et al., 2011; Scaramozza et al., 2014). Myotubes from ALS patients have a lower levels of fast, slow and neonatal myosin heavy chain (MHC) proteins when compared to control myotubes (Pradat et al., 2011), as well as decreased levels of the MyoG protein (Scaramozza et al., 2014). Muscle biopsies from ALS patients and healthy control have the same absolute number of satellite cells, as quantified by Pax7 staining. However, very few of these Pax7-positive nuclei are positive for MyoD, but surprisingly, positive for MyoG (Pradat et al., 2011). These observations in muscle cell culture and muscle tissue suggest that SCs appear to be moving into a senescent state and that there is a perturbation in the signals required for efficient and complete myogenesis. As a result, the skeletal muscle is unable to effectively repair and regenerate, resulting in severe muscle atrophy and weakness. However, the mechanisms contributing to these changes have not been precisely elucidated. Such knowledge is required before targeted therapies aimed at restoring myogenic capacity can be tested. Developing SC cultures from ALS patients is very challenging. Therefore, information relating to the impaired myogenic capacity in muscle cultures and muscle tissue has been obtained from a very small number of ALS patients. More studies are required to confirm and add to this knowledge base.

In the mutant SOD1^G93A^ (glycine to alanine mutation at position 93) ALS mouse impaired SCs number and activation is dependent on disease stage and muscle fiber type (Manzano et al., 2012). SC number is reduced in both slow (soleus) and fast (extensor digitorum longus; EDL) muscle fibers of SOD1^G93A^ mice compared to non-transgenic mice at the early presymptomatic stage (p40). However, fiber type differences appear with disease progression. At late presymptomatic stage (p60) the soleus muscle presented with an increased number of SCs, while there is no difference in the EDL muscle. At symptomatic stage (p90) the soleus muscle presented with a decreased number of SCs, while there was an increase in the EDL muscle. Finally, at end stage (p120), the SC count resembled that previously observed at p60, with an increased number of SCs in the soleus and no difference in the EDL muscle. The percentage of myofibre-associated SCs co-expressing Pax7 and MYOD was used as an indication of SC activation. At early presymptomatic stage (p40) no difference in SC activation was observed, however at p60 there was an increase and a tendency for a decrease in the soleus and EDL muscle, respectively (Manzano et al., 2012). At symptomatic stage (p90) there was an increase in the number of active SC in both fiber types, however this returned to the same levels as the non-transgenic mice at end stage (p120). These temporal and muscle-specific changes in SC number, and potential SC activation, are most likely indicative of an attempted adaptive response to regenerate the muscle due to toxic insults induced by mitochondrial dysfunction, oxidative stress, protein aggregation and denervation. As observed in muscle from ALS patients, an early dysregulation in MRFs may contribute to the alteration in SC number and SC activation. In presymptomatic (p60) mice *Pax7* and *Myog* mRNA and MYF5 protein were increased (Manzano et al., 2013). However, the upregulation of the Pax7 protein, as well as the mRNA levels of other MRFs, including *Myf5, Myod1*, and *Myog*, was not observed until mice were symptomatic (p90) (Manzano et al., 2013). While the changes in these mRNA levels were still visible at end stage (p120), there was no upregulation of the corresponding protein levels (Manzano et al., 2013).

While it is well established that skeletal muscle regeneration and size is significantly influenced by SC number and efficient activation of the various myogenic stages (Mauro, 1961), recent evidence has shown that SC's also play a role in maintaining NMJ health (Liu et al., 2015). Following nerve injury, SC's become active and divide during NMJ regeneration. SC depletion causes muscle fiber atrophy, increased connective tissue between the myofibres and impaired myofibre/NMJ connectivity. Interestingly, there was an increase in SC activity and fusion to existing myofibres in close proximity to where NMJ regeneration was occurring. As such, SC depletion resulted in inefficient reinnervation of the NMJ, impaired post-synaptic morphology and reductions in post-synaptic myonuclei. While the occurrence of this phenomenon has not been investigated in models of human ALS, it demonstrates the potential synergy between SC's and the NMJ. This further highlights the importance of maintaining optimal myogenesis in ALS.

## Skeletal muscle mitochondrial dysfunction in ALS

In SOD1^G93A^ ALS mice motor neuron mitochondrial degeneration precedes paralysis (Kong and Xu, 1998). As such, impaired mitochondrial function and death is considered an important component in ALS pathogenesis (Menzies et al., 2002). It has been well established in ALS that SOD1 mutations cause an accumulation of aggregates inside mitochondria, fragmented mitochondrial networks and mitochondrial depolarization and this has been reviewed extensively (Dupuis et al., 2004; Faes and Callewaert, 2011; Bozzo et al., 2016). In skeletal muscle of ALS patients and mice, impaired mitochondrial function is well known and reviewed previously (Appel, 2006; Shi et al., 2010). This is indicated by NADH:CoQ oxidoreductase and cytochrome c oxidase (COX) deficiency, reduced mitochondrial DNA and reduced levels of mitochondria Mn-SOD (Wiedemann et al., 1998; Vielhaber et al., 2000).

Abnormal skeletal muscle mitochondrial dynamics is also observed in presymptomatic SOD1^G93A^ ALS mice (Luo et al., 2013). As the disease progresses in SOD1^G93A^ ALS mice, SOD1 activity increases in skeletal muscle, but not in motor neurons, (Leclerc et al., 2001) suggesting that skeletal muscle is an important player in disease progression and severity. Overexpressing mutant SOD1^G93A^ in skeletal muscle of healthy mice produces similar perturbations in mitochondrial dynamics that are observed in ALS SOD1^G93A^ mice (Luo et al., 2013). This demonstrates that the skeletal muscle SOD1 mutation is sufficient to cause an ALS-like muscle pathology independent of any initial motor neuron degeneration and further supports the involvement of skeletal muscle in ALS pathogenesis.

Skeletal muscle mitochondria from SOD1 ALS mice also present with altered bioenergetics. Maximal oxygen consumption and ADP stimulated oxidative phosphorylation are lower in the soleus, but not the extensor digitorum longus (EDL) or diaphragm muscles, from 130 day old SOD1^G93A^ ALS mice, when compared with aged-matched controls (Leclerc et al., 2001). In the hindlimb muscles from SOD1^G86R^ ALS mice (glycine to arginine mutation at position 86), when compared with control mice, respiratory control ratio (ratio between state 3 and state 2 respiration) is decreased (Dupuis et al., 2003). This occurred without an associated reduction in COX activity, suggesting increased mitochondrial uncoupling. In parallel with this was the observation of lower ATP levels in the gastrocnemius muscle of the SOD1^*G*86*R*^ ALS mice at ages, 75, 90, and 105 days of age. Improvements in quadriceps muscle mitochondrial bioenergetics (in addition to spinal cord) in 90 day old SOD1^G93A^ ALS mice has been achieved by oral administration of the mitochondrial-targeted antioxidant [10-(4,5-dimethoxy-2-methyl-3,6-dioxo-1,4-cyclohexadien- 1-yl)decyl]triphenyl-, methanesulfonate (MitoQ). This intervention slowed the decline in mitochondrial respiratory function, improved NMJ stability, grip strength and prolonged survival by ~7 days in both male and female mice (Miquel et al., 2014).

In terms of potential molecular factors regulating skeletal muscle mitochondria in ALS, muscle samples from patients with FALS and SALS, as well as the SOD1^G93A^ ALS mouse model, exhibit a significant reduction in the mRNA and protein levels of the transcriptional co-activator, peroxisome proliferator-activated receptor-γ coactivator-1α (PGC-1α), when compared with age matched control subjects and nerve disease (ND) control patients (Russell et al., 2012; Thau et al., 2012). Up regulation of PGC-1α increases mitochondrial biogenesis and enhances mitochondrial function via the induction and activation of several nuclear transcription factors, such as nuclear respiratory factor-1 (NRF-1) (Wu et al., 1999) and estrogen-related receptor alpha (ERRα) (Schreiber et al., 2004) and several gene targets including mitofusin-2 (Mfn2) (Cartoni et al., 2005) and COX subunit IV (COX IV) (Puigserver et al., 1998). PGC-1α also attenuates muscle atrophy programs (Sandri et al., 2006). In addition to PGC-1α, several of its downstream effectors of mitochondrial function, NRF-1, ERRα, and COX IV, are also down regulated in FALS and SALS patients (Russell et al., 2012). Additionally, a decrease in PGC-1α in the spinal cord and motor cortex of post-mortem ALS patients and spinal cord of end stage ALS mice has also been observed (Thau et al., 2012). The elevation of PGC-1α has been attempted by crossing transgenic PGC-1α mice with various SOD1 ALS mouse models, but with varying results. Overexpression of PGC-1α in neurons of SOD1^G93A^ ALS mice improved motor function and survival (Zhao et al., 2011), while overexpressing PGC-1α in fast-twitch muscles of SOD1^G37R^ (glycine to arginine mutation at position 37) ALS mice improved skeletal muscle function and mitochondrial biogenesis, but did not preserve neurodegeneration or extend survival (Da Cruz et al., 2012). However, in transgenic SOD1^G37R^/PGC-1α mice that are born with elevated PGC-1α levels, the transgenic advantage is lost as they move from symptomatic to end-stage. By end-stage their PGC-1α levels are reduced to those of the ALS wild-type mice (Da Cruz et al., 2012). This suggests the existence of a potent post-transcriptional regulatory mechanism that reduces PGC-1α levels in ALS.

## Mitochondrial dysfunction and the neuromuscular junction

Skeletal muscle fibers from 37 day old pre-symptomatic SOD1^G93A^ ALS mice have a reduction in mitochondrial inner membrane potential in the fiber segments proximal to the NMJ (Zhou et al., 2010). These fiber segments also had greater osmotic stress-induced Ca^(2+)^ release activity that were confined to regions of depolarized mitochondria. It is therefore of interest to investigate if this may contribute to NMJ degeneration. Of note, the loss of skeletal muscle mitochondrial membrane potential and disturbed Ca^2+^ handling in these 37 day old SOD1^G93A^ ALS mice occurs earlier than defects previously detected in motor neuron terminals from 47 day old SOD1^G93A^ ALS mice (Kennel et al., 1996; Fischer et al., 2004). This further supports the notion that skeletal muscle plays a direct role in disease on set and progression. Studies using Sod1^(−/−)^ mice to investigate the relationship between muscle mass, mitochondrial function and NMJ degeneration have reported interesting findings. Global Sod1^(−/−)^ mice have muscle atrophy associated with a progressive decline in mitochondrial bioenergetics and an increase in the production of mitochondrial reactive oxygen species (ROS) (Jang et al., 2010). Interestingly the these global Sod1^(−/−)^ mice also have an increase in muscle mitochondrial content near the NMJ. However, these mitochondria have impaired function that is associated with increased denervation of the NMJs and fragmented acetylcholine receptors. In contrast, skeletal muscle specific Sod1^(−/−)^ mice have normal ATP production and do not exhibit significant muscle atrophy, NMJ degeneration or increases in oxidative ROS production, although muscle contractile force is reduced (Zhang et al., 2013). These results suggest that a loss of Sod1 activity, specific to skeletal muscle, does not cause mitochondria dysfunction that is sufficient to activate muscle and NMJ degeneration pathways. In contrast, whole body loss of Sod1 presents a more dramatic phenotype where by its central and peripheral ablation may converge to severely impact skeletal muscle health. While these studies provide interesting insights into the direct and indirect roles of Sod1, the direct relevance of Sod1 knock-out mice to ALS pathology needs to be considered with caution. The mutations in SOD1 observed in ALS does not result in its ablation, but instead its accumulation and gain-of-function (Tsuda et al., 1994). The consequences of SOD1 mutations appears different to Sod1 ablation, especially in skeletal muscle.

As mitochondrial dysfunction is seen as key factor in ALS pathogenesis, targeted interventions have been used to try and improve mitochondrial function and calcium handling. Various approaches used include intrathecal administration of the immunosuppressant cyclosporine A (Keep et al., 2001), ablation of the mitochondrial matrix protein cyclophilin D (Martin et al., 2009; Parone et al., 2013) and treatment with GNX-4728, a modulator of the mitochondrial permeability transition pore (Martin et al., 2014). These treatments all improved mitochondrial function and survival with improvement in calcium handling (Parone et al., 2013) and NMJ stability (Martin et al., 2014).

Mice overexpressing uncoupling protein-1 (UCP1) in skeletal muscle have decreased mitochondrial membrane potential and respiratory control ratio (Dupuis et al., 2009) that is associated with muscle atrophy, a shift from a fast to slow muscle fiber type phenotype and a deterioration in the NMJ; alterations also observed in ALS mice (Gordon et al., 2010). SOD1^G86R^ ALS mice crossed with the muscle specific UCP1 transgenic mice have a more severe disease progression that coincides with NMJ deterioration. Further evidence linking impaired mitochondrial function with NMJ deterioration comes from mice manipulated with the ubiquitously expressed mitochondrial-targeted endonuclease, mito-PstI, that develop double-strand breaks in their mitochondrial DNA (Wang et al., 2013). These mice have muscle wasting and reduced locomotor activity later in life. This coincided with a decline in muscle SC number and muscle regenerative capacity. NMJ integrity was also compromised as indicated by impaired acetylcholinesterase (AChE) activity and development.

While more direct experimental studies are still required to establish cause and effect there are now several studies that demonstrate associations between skeletal muscle mitochondrial dysfunction, SC activation and NMJ stability. It appears that effective cross talk between mitochondria, SC's and the NMJ is required to maintain optimal skeletal muscle size and function and that an impairment in this signaling may have detrimental consequences, contributing to an ALS-like phenotype.

## microRNA (miRNA) regulation in ALS skeletal muscle

MiRNAs are small, non-coding RNAs that post-transcriptionally regulate gene expression and inhibit protein translation (Bartel, 2004), although in some instances they can increase gene expression (Vasudevan et al., 2007). Few studies have investigated the role of skeletal muscle miRNAs in the regulation of myogenesis, mitochondrial biogenesis and NMJ innervation in ALS. Muscle-enriched miRNAs (myomiRs) such as miR-1, miR-133, and miR-206 regulate SC proliferation and differentiation *in vitro* (Chen et al., 2006). miR-1 promotes myogenesis by inhibiting histone deacetylase 4 (HDAC4), a transcriptional repressor of muscle gene expression. On the other hand, miR-133 induces SCs proliferation by repressing serum response factor (SRF). Similarly, miR-206 increases SC proliferation and differentiation by repressing Pax3 and Pax7 protein levels (Kim et al., 2006; Chen et al., 2010; Dey et al., 2011). Additionally, miR-489 (Dey et al., 2011), miR-27b (Crist et al., 2009), and miR-181 (Naguibneva et al., 2006) also downregulate Pax7, Pax3, and homeobox protein Hox-A11, respectively, resulting in the promotion of SC differentiation. Overexpression of miR-125b, inhibits myoblast differentiation in culture and muscle regeneration in mice via its inhibition of IGF-2. miR-128 also inhibits bovine SC proliferation and myogenic differentiation via suppressing specificity protein-1 (Sp1) (Dai et al., 2016). In contrast, inhibition of miR-128 increases Sp1 protein levels resulting in a suppression of proliferation and an increase in differentiation. The miR-128/Sp1 regulatory axis influences myogensis as Sp1 is required to activate MyoD and supresses cyclin-dependent kinase inhibitor 1A (CDKN1A). In ALS, SC activation, proliferation and differentiation are in state of disequilibrium due to the intrinsic stress placed on the skeletal muscle. Investigating the regulation of these miRNAs and their known gene targets may provide a better understanding of how to combat the myogenic perturbations associated with ALS.

In muscle samples from patients with ALS, when compared with healthy controls, elevated levels of miR-206, miR-23a, miR-29b, miR-31, and miR-455 was observed (Russell et al., 2012). miR-1 and 181 levels were elevated, but not statistically significant; potentially due to the relatively small sample size. More studies are required to extend and validate these observations in human ALS muscle. Similarly, in muscle from SOD1^G93A^ ALS mice increases greater than 2-fold were observed for several miRNAs, including miR-1, miR-133, miR-206, miR-23a, and miR-29a (see online Supplementary Figure S1 in Williams et al., 2009). Whether the regulation of these miRNAs is a cause of impaired myogenesis or an attempt to rescue this process in ALS is presently unknown.

miR-23a is another miRNA dysregulated in skeletal muscle of ALS patients. In skeletal muscle from ALS patients, the increase in miRNA-23a was associated with a decrease in PGC-1α mRNA and protein level (Russell et al., 2012). miRNA-23a is a direct inhibitor of PGC-1α *in vitro* (Russell et al., 2012) and mice over expressing miR-23a have a reduction in PGC-1α levels (Wada et al., 2015). In ALS the elevated levels of skeletal muscle miR-23a may play a role in the dysregulation of skeletal muscle mitochondria and the NMJ, however this direct relationship has not yet been investigated.

In SOD^G93A^ ALS mice, miR-206 is dramatically upregulated in skeletal muscle (Williams et al., 2009). It appears that the upregulation of miR-206 is a stress-related response to the ALS disease, as ALS mice rendered deficient in miR-206 have an accelerated disease progression. miR-206 plays a compensatory role by promoting the regeneration of neuromuscular synapses and slowing the ALS disease progression. The effect of miR-206 on NMJ regeneration is partly explained by its regulation of HDAC4 and FGF signaling. miR-206 also regulates other SCs markers including connexin 43 (Cx43) (Araya et al., 2005), utrophin (Utrn), follistatin-like 1 (Fstl-1) (Rosenberg et al., 2006), and MyoD (Dey et al., 2011; Koning et al., 2012). miR-206 upregulation in ALS (Williams et al., 2009) may also be an attempt to positively affect SC activation, proliferation and differentiation required for myogenesis, as well as assist with NMJ protection. However, this requires experimental validation.

## Conclusion

There is now a large body of evidence supporting the role of skeletal muscle impairments in the development and severity of ALS. The dysfunction in skeletal muscle SC activation, proliferation and differentiation, as well as mitochondrial function, may synergize to develop a less than optimal environment for the maintenance of NMJ innervation. Consequently, this may instigate retrograde signaling through the NMJ and contribute to the degeneration of motor neurons. The precise cause of these perturbations are unclear. From a molecular level, alterations in the transcriptional and post-translational control of key genes/proteins involved in myogenesis and mitochondrial biogenesis have been postulated. Biochemically, impaired Ca^2+^ handling and ROS removal may contribute to the development of a toxic muscle milieu. Importantly, many of these skeletal muscle perturbations, most notably in the mitochondria and NMJ, occur in the presymptoimatc stage. These observations suggest that skeletal muscle plays a direct role in disease on set, progression and severity. Studies targeting skeletal muscle that are aimed at understanding the interrelationship between SC activation, myogenesis, mitochondrial function and NMJ health in ALS, may contribute to better understanding the disease etiology as well as its progression. In doing so this research may identify novel mechanisms contributing to disease on set, as well as novel targets that may lend themselves to therapeutic manipulation.

## Author contributions

All authors listed, have made substantial, direct and intellectual contribution to the work, and approved it for publication.

### Conflict of interest statement

The authors declare that the research was conducted in the absence of any commercial or financial relationships that could be construed as a potential conflict of interest.
